# *Actinomyces* Produces Defensin-Like Bacteriocins (Actifensins) with a Highly Degenerate Structure and Broad Antimicrobial Activity

**DOI:** 10.1128/JB.00529-19

**Published:** 2020-01-29

**Authors:** Ivan Sugrue, Paula M. O’Connor, Colin Hill, Catherine Stanton, R. Paul Ross

**Affiliations:** a School of Microbiology, University College Cork, Cork, Ireland; b Teagasc Food Research Centre, Fermoy, Ireland; c APC Microbiome Ireland, Cork, Ireland; Brigham and Women's Hospital/Harvard Medical School

**Keywords:** *Actinomyces*, bacteriocin, defensin, antimicrobial peptide, actifensin

## Abstract

We identified a strain of Actinomyces ruminicola which produces a potent bacteriocin with activity against a broad range of Gram-positive bacteria, many of which are pathogenic to animals and humans. The bacteriocin was purified and found to have a mass of 4,091 ± 1 Da with a sequence of GFGCNLITSNPYQCSNHCKSVGYRGGYCKLRTVCTCY containing three disulfide bridges. Surprisingly, near relatives of actifensin were found to be a series of related eukaryotic defensins displaying greater than 50% identity to the bacteriocin. A pangenomic screen further revealed that production of actifensin-related bacteriocins is a common trait within the genus, with 47 being encoded in 161 genomes. Furthermore, these bacteriocins displayed a remarkable level of diversity with a mean amino acid identity of only 52% between strains/species. This level of redundancy suggests that this new class of bacteriocins may provide a very broad structural basis on which to deliver and design new broad-spectrum antimicrobials for treatment of animal and human infections.

**IMPORTANCE** Bacteriocins (ribosomally produced antimicrobial peptides) are potential alternatives to current antimicrobials given the global challenge of antimicrobial resistance. We identified a novel bacteriocin from Actinomyces ruminicola with no previously characterized antimicrobial activity. Using publicly available genomic data, we found a highly conserved yet divergent family of previously unidentified homologous peptide sequences within the genus *Actinomyces* with striking similarity to eukaryotic defensins. These actifensins may provide a potent line of antimicrobial defense/offense, and the machinery to produce them could be used for the design of new antimicrobials given the degeneracy that exists naturally in their structure.

## Abstract (precis)

Bacteriocins (ribosomally produced antimicrobial peptides) are potential alternatives to current antimicrobials given the global challenge of antimicrobial resistance. We identified a novel bacteriocin from Actinomyces ruminicola with no previously characterized antimicrobial activity. Using publicly available genomic data, we found a highly conserved yet divergent family of previously unidentified homologous peptide sequences within the genus *Actinomyces* with striking similarity to eukaryotic defensins. These actifensins may provide a potent line of antimicrobial defense/offense, and the machinery to produce them could be used for the design of new antimicrobials given the degeneracy that exists naturally in their structure.

## INTRODUCTION

Novel antimicrobial compounds are increasingly important in the food, agriculture, and medical fields due to decreasing efficacies of current antimicrobial treatments. Bacteriocins are ribosomally synthesized antimicrobial peptides produced by bacteria which can target another bacterium of the same species (narrow spectrum) or bacteria of other species/genera (broad spectrum) (1). Bacteriocin producers are self-protected through the production of specific immunity proteins, and as bacteriocins are gene encoded, they can be genetically modified. Bacteriocins produced by Gram-positive bacteria have been grouped according to their primary structure into class I (posttranslationally modified bacteriocins) and class II (unmodified or cyclic bacteriocins) (2). Class II is split into several subgroups, including the class IId bacteriocins, which are a heterogenous group of linear, unmodified, nonpediocin-like peptides (3).

Defensins are antimicrobial peptides ubiquitous among eukaryotes which play a role in innate immunity but have also been found to act as signaling peptides, toxins, enzyme inhibitors, and abiotic stress responders and to have anticancer properties. Defensins are small (<10 kDa) cysteine-rich (forming three to six disulfide bonds) peptides with low amino acid identity, and the two superfamilies are thought to have evolved convergently (4). Only two expressed defensin-like bacteriocins have been described; the laterosporulins were previously identified among prokaryotes and contain disulfide bonds in positions homologous to those in eukaryotic defensins (5, 6). Other disulfide bond-containing bacteriocins, such as bactofencin, have been compared with eukaryotic defensins due to their highly cationic nature (7, 8). Laterosporulin and its homolog laterosporulin10 are class IId bacteriocins produced by *Brevibacillus* spp. which have been described as broad-spectrum antimicrobials against both Gram-negative and Gram-positive bacteria. The two peptides are 5.6 kDa and 6.0 kDa and share only 57.6% amino acid sequence identity but have conserved cysteines, which are characteristic of eukaryotic defensins (6).

*Actinomyces* spp. are a heterogenous group of high-GC-content, Gram-positive non-spore-forming facultative or obligate anaerobes that belong to the *Actinomycetaceae* family within the phylum *Actinobacteria* (9). In humans, a number of species are known colonizers of hard surfaces in the oral cavity, where they play a key role in plaque biofilm formation (10, 11). They have been identified as core members of the oral bacteriome, present in moderate abundance (>0.1% to >2.0%) among geographically diverse populations (10, 12–15). *Actinomyces* spp. have been implicated in oral health as being associated in greater abundance in individuals with dental caries, one of the most prevalent chronic oral diseases worldwide (14, 15). Most characterized strains are clinical isolates of human origin, while some opportunistically pathogenic species such as Actinomyces israelii and Actinomyces gerencseriae are known to cause the uncommon infectious disease actinomycosis (16). Though *Actinomyces* spp. are abundant in the oral cavity, little is known about their presence in the gut, probably due to their low abundance (<0.1%) (10). Many *Actinomyces* spp. have been isolated from fecal material and from the gastrointestinal tracts of different animals, indicating a propensity for gastric transit survival, and their presence has also been noted in the urogenital tract (17–24). Here, we identify a new group of bacteriocins using a pangenomic *in silico* approach paired with functional screening. Many *in silico* genome mining tools have been developed for the successful detection of novel antimicrobial-producing operons (25, 26). Obviously, these methods rely on relationships with previously known genes; therefore, functional screening is crucial for the identification of unrelated antimicrobials. In this study, we isolated a potent bacteriocin-producing strain of Actinomyces ruminicola from sheep feces; the bacteriocin produced resembled eukaryotic defensins, having three characteristic disulfide bridges. A subsequent pan-genus *Actinomyces* analysis revealed that such bacteriocins are widely distributed in these bacteria, albeit with a highly variable structure.

## RESULTS

### Identification of a novel bacteriocin-producing *Actinomyces* sp.

**FIG 1:**
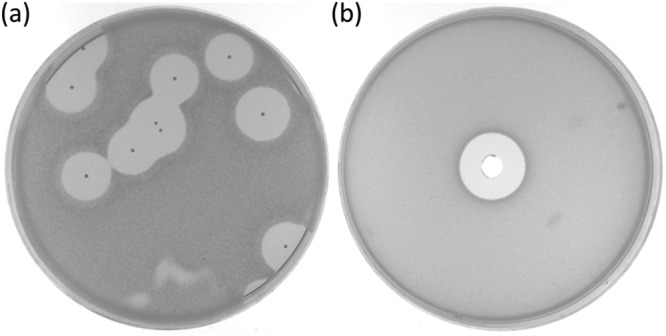
Antimicrobial activity of Actinomyces ruminicola DPC 7226 from colonies overlaid with L. delbrueckii subsp. bulgaricus LMG 6901 in sloppy MRS (a) and in well diffusion with neutralized CFS (b).

Actinomyces ruminicola DPC 7226 was isolated from sheep feces. During an initial screen of >10,000 colonies for bacteriocin producers, this strain was found to produce a large zone of inhibition when overlaid with an acid-tolerant indicator species, Lactobacillus delbrueckii subsp. *bulgaricus* LMG 6901 (Fig. 1a). The neutralized cell-free supernatant (CFS) was also found to produce a zone of inhibition against *L. delbrueckii* subsp. *bulgaricus* LMG 6901, indicating production of a soluble antimicrobial molecule (Fig. 1b). This activity was eliminated when the supernatant was treated with proteinase K, demonstrating that the antimicrobial is proteinaceous in nature (data not shown).

**FIG 2:**
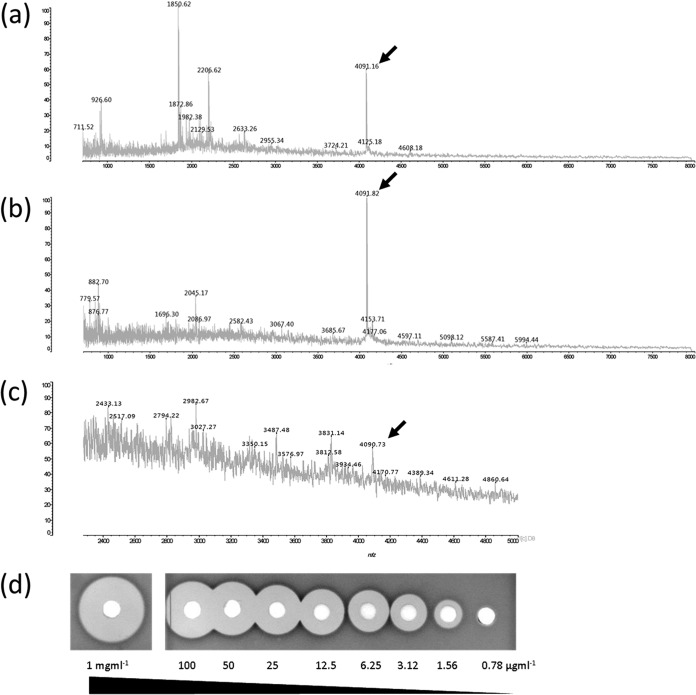
Detection of actifensin 4,091 Da ± 1 Da (indicated by arrows) by MALDI-TOF MS from cell-free supernatant (a), cell extract (b), and colonies on a plate (c). (d) The 4,091 (±1)-Da compound when purified was active to <1 μg · ml^−1^; indicator, *L. bulgaricus* LMG 6901.

Antimicrobial activity was purified from pelleted bacterial cells (C_18_ SPE; reversed-phase high-performance liquid chromatography [HPLC]) and CFS (Amberlite XAD, C_18_ SPE; reversed-phase HPLC), and matrix-assisted laser desorption ionization–time of flight mass spectrometry (MALDI-TOF MS) of active peaks detected a mass of 4,091 ± 1 Da (Fig. 2a and b). The mass was also detected by colony MS (Fig. 2c). The activity of the HPLC-purified fraction from CFS was assayed against *L. delbrueckii* subsp. *bulgaricus* LMG 6901 and found to be active at <1 μg · ml^−1^ (Fig. 2d). The antimicrobial peptide was found to be heat stable, retaining almost all activity after treatment for 30 min at 100°C, but was completely lost after treatment at 121°C for 15 min.

### Spectrum of inhibition.

**FIG 3:**
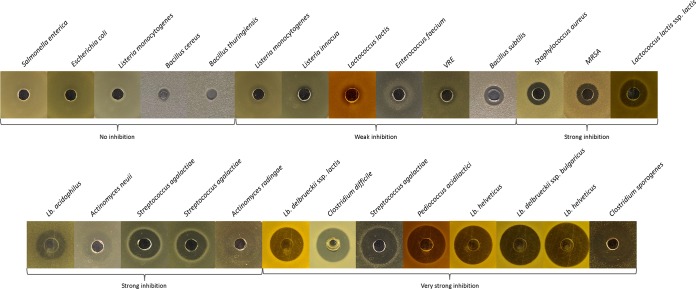
Inhibition of actifensin against a broad spectrum of indicator species. Weak inhibition, 0.5- to 3-mm zone; strong inhibition, 3- to 5-mm zone; very strong inhibition, >5-mm zone. VRE, vancomycin-resistant *Enterococcus*; MRSA, methicillin-resistant Staphylococcus aureus.

A range of indicator organisms was tested against the purified antimicrobial to determine the spectrum of inhibition. The antimicrobial was active against a broad range of genera, with 22 of the 27 strains screened inhibited to various degrees, including species of the genera *Lactococcus*, *Enterococcus*, *Lactobacillus*, *Streptococcus*, *Pediococcus*, *Bacillus*, *Staphylococcus*, other *Actinomyces* spp., and *Clostridium* spp. (Fig. 3). No inhibition against the Gram-negative species Salmonella enterica or Escherichia coli was observed. *Listeria* spp. and *Bacillus* spp. were inhibited weakly or not at all (Fig. 3). Inhibition against other *Actinomyces* spp. was found, and activity was particularly strong against Staphylococcus aureus and Clostridium difficile.

MICs were determined against Enterococcus faecium APC1031, E. faecium NCDO0942, S. aureus R693, Streptococcus agalactiae APC1055, and C. difficile DPC6534 (see Fig. S1 in the supplemental material). Enterococci were inhibited at 3.05 to 6.1 μM. S. aureus was inhibited at 3.05 μM. S. agalactiae and C. difficile were inhibited at 0.76 μM (Fig. S1).

### Distribution of genes encoding bacteriocins in the genus *Actinomyces*.

As the active mass could not be matched to any previously known antimicrobial peptide and no antimicrobial compounds were previously described within the species, the genome of *A. ruminicola* DPC 7226 was sequenced. Following genome annotation, the draft genome was analyzed using BAGEL4 to search for potential antimicrobial-encoding operons. Gene clusters were identified containing putative genes for thiopeptide production (data not shown), but the masses predicted, 2,195.4 Da and 1,152.5 Da, did not correspond with the mass detected in the antimicrobial HPLC fraction.

**FIG 4:**
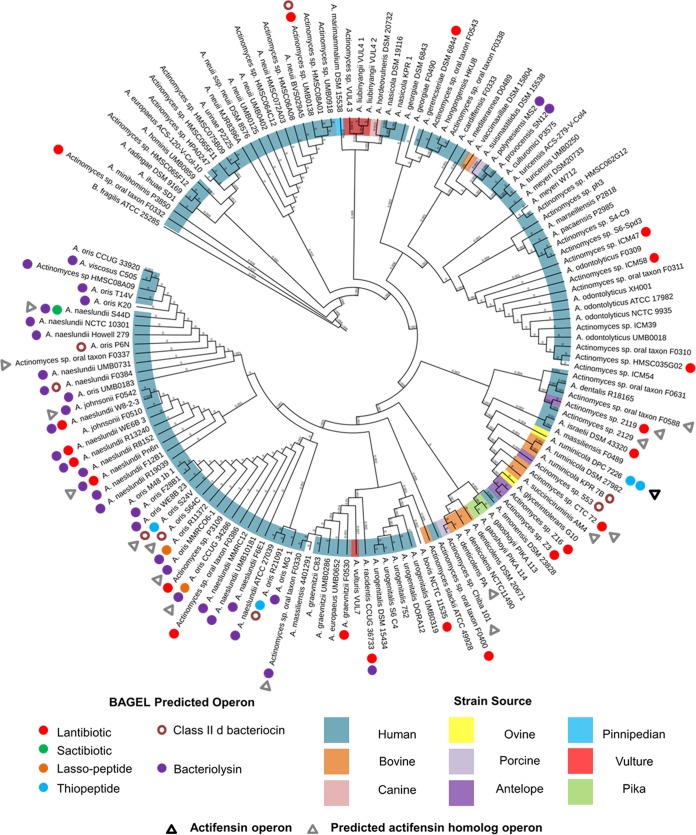
Phylogram of *Actinomyces* genomes using 16S sequences overlaid with BAGEL4 predictions, strain source, and presence of actifensin or predicted homolog operon.

In conjunction with screening of the genome of *A. ruminicola* DPC 7226, we also set out to characterize the antimicrobial potential of the genus. One hundred sixty-one *Actinomyces* species genomes in various stages of assembly were screened using BAGEL4 (see Table S1). The isolates were obtained from humans (78.2%) or other animals (16.1%) or were of unknown origin (4.9%), while one was an environmental isolate (0.6%). One hundred six areas of interest were revealed in 76 strains, covering 18 species. Ninety areas of interest contained complete operons for antimicrobial production. Twenty-nine were predicted to encode class I bacteriocins, including 7 LanBC modified lantibiotics, 16 LanM modified lantibiotics, 1 single-peptide sactibiotic, 3 lasso peptides, and 2 thiopeptides. Thirteen operons were predicted to encode class IId bacteriocins, and a further 48 operons were predicted to encode bacteriolysins. A phylogenetic tree was generated from the 16S rRNA sequences of 142 *Actinomyces* genomes with Bacteroides fragilis ATCC 25285 as the root and overlaid with operon type and strain source (Fig. 4). Bacteriocin production was widely distributed across the *Actinomyces* pangenome, though bacteriolysin production was found exclusively among human isolates (Fig. 4).

### Genetic and molecular characterization of the actifensin determinant.

**FIG 5:**
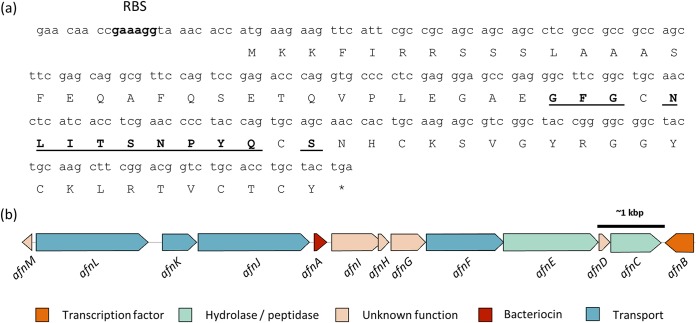
(a) Sixty-nine-residue propeptide identified following genome analysis using the 15-amino-acid sequence (underlined) determined by N-terminal amino acid sequencing. RBS, putative ribosome binding site highlighted 8 bp upstream of the start codon. (b) Genetic vicinity of structural gene containing nearby genes for transport, hypothetical and proteolytic proteins, and a transcription factor.

To identify the gene encoding the 4,091 (±1)-Da peptide within the genome of *A. ruminicola* DPC 7226, pure peptide was subjected to N-terminal sequencing, which revealed a primary sequence consisting of Gly-Phe-Gly-X-Asn-Leu-Ile-Thr-Ser-Asn-Pro-Tyr-Glu-X-Ser, with blanks at residue positions 4 and 14 denoted as probable cysteines (Fig. 5a). This 15-amino-acid sequence was matched to a 69-residue small open reading frame in the draft genome, capable of encoding a 37-amino-acid mature peptide (hereafter referred to as actifensin) with a predicted mass of 4,097.7 Da preceded by a 32-residue leader sequence (Fig. 5a).

The genetic locus encoding actifensin is shown in Fig. 5b, where *afnA* encodes actifensin. Within an approximately 6.5-kbp upstream region of *afnA*, genes encoding an ABC transporter permease (*afnJ*), an ATP binding ABC transporter (*afnK*), and another ABC transporter permease (*afnL*) were identified as being present. Downstream of *afnA*, three hypothetical genes of unknown function (*afnG* to *afnI*) were found, followed by genes encoding another ATP binding ABC transporter (*afnF*), a predicted α/β hydrolase superfamily protein (*afnE*), another protein of unknown function, a subtilisin-like protease, and a LuxR family transcription factor (*afnD*, *afnC*, and *afnB*, respectively). Within *afnE* is a predicted RHO-independent transcription terminator, and upstream of the structural gene are four predicted promoters. A putative ribosome binding site was also identified nine base pairs upstream of the ATG start codon for the peptide consisting of a purine rich sequence, 5′-GAAAGG-3′ (Fig. 5a).

The leaderless structural peptide was found to have a predicted mass of 4,097.7 Da. This mass was approximately 6 Da higher than detected by MALDI-TOF MS. The difference between predicted and observed masses most likely corresponds to the loss of six hydrogen atoms during the formation of disulfide bonds between the six cysteines. Short peptides with numerous disulfides in specific positions are characteristic of the defensin peptide families (4). To confirm the presence of disulfide bonds in actifensin, pure peptide was reduced and alkylated to break open the disulfide bonds and then subjected to trypsin digestion and peptide mass fingerprint analysis by MALDI-TOF MS. Reduction and alkylation of actifensin resulted in a 4,440-Da mass, which correlates with the expected increase in mass of 58 Da for each cysteine. MALDI-TOF MS analysis of the subsequent trypsin digest detected a mass of 2,257.02 Da, which corresponds to the first 19 amino acids of the peptide (Gly-1 to Lys-19) containing three alkylated cysteine residues. Three other predicted masses for Ser-20 to Arg-24, Gly-25 to Arg-31, and Thr-32 to Tyr-37 (predicted and alkylated masses of 581.30 Da, 584.25 Da, and 803.31 Da, respectively) were not detected.

### Discovery of actifensin homologs.

**FIG 6:**
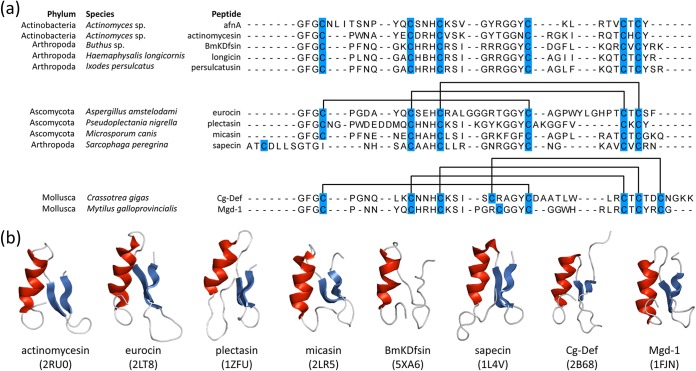
(a) Mature peptide sequence alignment of AfnA with characterized defensin family peptides from different phyla. Known disulfide connectivities are outlined above highlighted cysteine residues. (b) Available 3D structures of sequences in panel a. Alpha helices are colored red, and beta sheets are shown in blue. Protein data bank accession numbers shown below the structres (in parentheses).

**FIG 7:**
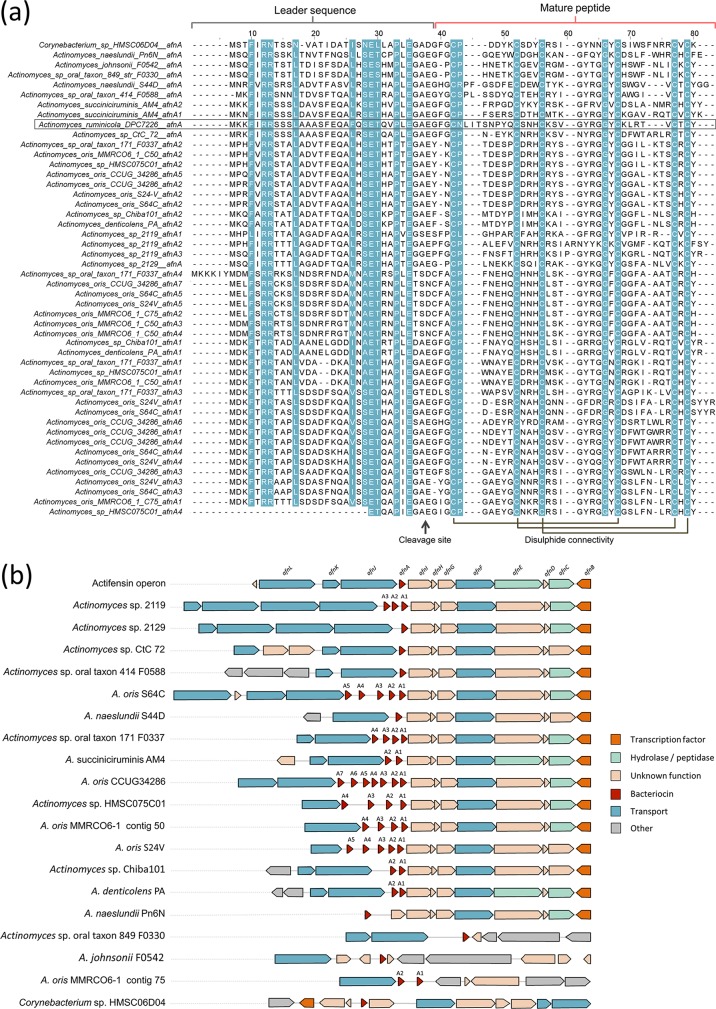
(a) Sequence alignment of actifensin propeptide sequence (boxed) with structural genes predicted for *Actinomyces* sp. peptides. Amino acids with greater than 80% conservation are colored, and leader sequences and mature active peptides are indicated at the top. Putative disulfide connectivity between conserved cysteines of the mature peptide is indicated at the bottom right, and putative cleavage sites are indicated at the bottom center. (b) Diagrams of actifensin homolog production operons. Multiple bacteriocin genes within one operon are denoted *afnA1* to *afnA7* where present.

BLASTp analysis with AfnA found homologous open reading frames (ORFs) within the fungal genera *Blastomyces*, *Emmonsia*, and *Emergomyces*, Helicocarpus griseus, and a defensin from the mollusk species Ruditapes philippinarum (58%, 58%, 55%, 52%, and 61% identity, respectively) (see Fig. S2). Characteristic conserved cysteines were noted, though low sequence identity was observed between the mature actifensin peptide and eukaryotic defensins. The same was found when AfnA was compared with known previously characterized arthropod, ascomycete, and mollusk defensins (Fig. 6a) with conserved secondary structures (Fig. 6b). BLASTp analysis using the 69-residue AfnA sequence identified 37 homologous structural genes within the genus *Actinomyces* and one homolog from a *Corynebacterium* sp. sequence (Fig. 7a). Further analysis indicated that the homologs were present in 15 operons from 14 strains, in addition to conserved genes for transport, transcription regulation, and proteolytic activity (Fig. 7b). *Actinomyces* sp. strain 2119, Actinomyces oris S64C, Actinomyces succiniciruminis AM4, *A. oris* CCUG34286, *Actinomyces* sp. strain F0337, *Actinomyces* sp. strain HMSC075C01, and *A. oris* MMRCO6-1 had at least two actifensin homologs, while *Actinomyces* sp. F0337 contained an operon with seven copies, the most observed within one genome (Fig. 7b). The genome of *A. oris* MMRCO6-1 contained six encoded actifensin homologs detectable over two contigs, but only one (contig 50) contained the other conserved ORFs (*afnB-I* and *afnJ-K*) present in the actifensin operon. Twelve of 14 operons had a highly conserved arrangement of *afnB-I*, all of which also had ABC transporter genes directly upstream of the bacteriocin ORF. The mean amino acid identity between all structural genes was 52%. The highest identity observed between actifensin and a homolog was 77% identity with *afnA* in *Actinomyces* sp. strain CTC72, though higher identities were observed between other peptides (see Fig. S3). We proceeded to characterize ten predicted cysteine-stabilized αβ (CSαβ) peptides predicted by Dash et al. (27). The peptides are present in five *Actinomyces* genomes bringing the total number of peptides to 47 homologous structural genes in 19 strains. Actinomyces oris S24V, Actinomyces denticolens PA, *Actinomyces* sp. strain Chiba-101, Actinomyces johnsonii F0542, and *Actinomyces* sp. strain F0330 have genes which were not identified using BLASTp and the actifensin propeptide sequence (27). Strains S24V, PA, and Chiba-101 display the conserved *afnB* to *afnI* ORFs following *afnA*, which are absent in strains F0330 and F0542 (Fig. 7b).

The propeptide contains a conserved G-X-E motif prior to the start of the mature peptide (Fig. 7a). In 36 of the peptides, an alanine residue is present after the glycine, which may be involved in secretion and cleavage. This putative GA cleavage signal is replaced by a TS motif in 8 of the 49 peptides (*A. oris* S64C *afnA5*, *A. oris* CCUG34286 *afnA7*, *A. oris* MMRCO6-1 contig 75 *afnA2*, *Actinomyces* sp. F0337 *afnA4*, *Actinomyces* sp. HMSC075C01 *afnA4*, *A. oris* MMRCO6-1 contig 50 *afnA4* and *afnA3*, and *A. oris* S24V *afnA5*). A conserved Pro residue was noted following the first conserved Cys in addition to a conserved G-Y-X-G-G-X-C sequence at positions 56 to 62 of the propeptide (22 to 28 in the active peptide) (Fig. 7a).

## DISCUSSION

We describe a novel group of bacteriocins with broad-spectrum inhibitory activity within the *Actinomyces* genus. Actifensin is the first such bacteriocin to be discovered, which is produced by a strain of Actinomyces ruminicola.

Actifensin inhibited a broad range of Gram-positive species, including notable pathogens such as vancomycin-resistant *Enterococcus* and methicillin-resistant *Staphylococcus*. Given the global challenge of the increase in antibiotic resistance, there is an urgent need for new classes of antimicrobials. Bacteriocins have been suggested as an alternative to conventional antibiotics due to their effectiveness at low concentrations and their potential to be genetically modified (2). Class II bacteriocins are diverse in sequence and structure whose mechanism of action is through interaction with the cell membrane, causing permeabilization and pore formation and dissipating the membrane potential (3). The defensin-like bacteriocin laterosporulin10 has been found to act on the cell membrane of S. aureus Mtb H37Rv, disrupting cellular homeostasis (6). Plectasin and eurocin, fungal C6 defensins, are known to bind lipid II, inhibiting bacterial cell wall biosynthesis (45, 46). Actifensin possesses an N-terminal loop extension which, in other defensin peptides, has been implicated in membrane disruptive capability (31). The loop consists of nine residues between Cys-4 and Cys-14 beginning with an Asn. In most of the other peptide sequences identified, the N loop is six residues long, beginning with a Pro (except in AfnA from *Actinomyces* sp. strain F0588 or *A. naeslundii* S44D, which has an eight-residue N loop with a serine or arginine in the first position, respectively, followed by a Pro) (Fig. 7a).

Actifensin also inhibited the growth of C. difficile and Clostridium sporogenes. Clostridia are known colonizers of the rumen, and as *A. ruminicola* DPC7226 was isolated from the feces of a ruminant, actifensin production may provide a competitive advantage in the gut microbiome. Actinomyces neuii and Actinomyces radingae were both inhibited by actifensin; however, it would be interesting to see if cross-resistance between actifensin and other actifensin-like producers exists.

A pangenus *in silico* screen revealed that the genus *Actinomyces* (Fig. 4) is a rich source of antimicrobials and has genes for bacteriolysin and lantibiotic production (48/90 and 29/90 operons, respectively). Thirteen class II bacteriocins were predicted by BAGEL, but neither the actifensin operon nor its homologs were detected due to lack of similarity with known systems. One previous study described odontolycin, a bacteriocin produced by an Actinomyces odontolyticus dental plaque isolate, though no further research on the peptide was reported (34). Interestingly, in our study, no operons for bacteriocin production were found among five *A. odontolyticus* genomes screened (Fig. 4).

The actifensin structural gene encodes a 37-amino-acid mature peptide preceded by a 32-amino-acid leader sequence (Fig. 5). A GA motif at positions −3 and −2 was identified, which is a known cleavage signal used in ABC transporter-mediated secretion (36). Indeed, there are a number of predicted ABC transporter genes within the actifensin operon. ABC transporter genes could also play a role in self-immunity to the actifensin peptide. Unusually, an additional glutamic acid residue is present at position −1 before the mature peptide. As the purified peptide was subjected to N-terminal sequencing, we can be certain that the mature peptide begins with a glycine residue. Therefore, the additional glutamic acid residue at position −1 is most likely subject to exopeptidase cleavage prior to activity, and indeed, there are genes present with predicted protease activities (Fig. 5).

The GA cleavage motif is present in 36 of the homolog structural genes, with TS replacing the motif in eight instances, GT and GG in two cases, and GS, SA, and DA in one each (Fig. 7a). A double glycine is the most commonly found motif for ABC transporter-mediated cleavage among bacteriocins, though GA and GS have also been observed (36). It will be interesting to see if the peptides bearing other residues at this location are indeed subject to ABC-mediated transport. We note that each operon containing a gene with a nontraditional TS/GT/SA/DA signal contains at least one more structural gene than those with a GG/GA sequence. This could indicate potential diversification of a repertoire of bacteriocins enabling improved ability to combat multiple competitors. It was also surprising that an actifensin homolog was found in a distantly related *Corynebacterium* sp., though many of the conserved genes in the *Actinomyces* sp. operons were not present (Fig. 7b). As such, this may be nonfunctional, as ABC transporter-related genes are missing upstream of the structural gene and the conserved *afnB* to *afnI* pattern is absent. The genera *Corynebacterium* and *Actinomyces* are distantly related members within the phylum *Actinobacteria*, and some species are known members of plaque biofilms, providing an opportunity for horizontal gene transfer (16). However given the dissimilarity of the operons, they may have been acquired independently at some stage.

**FIG 8:**
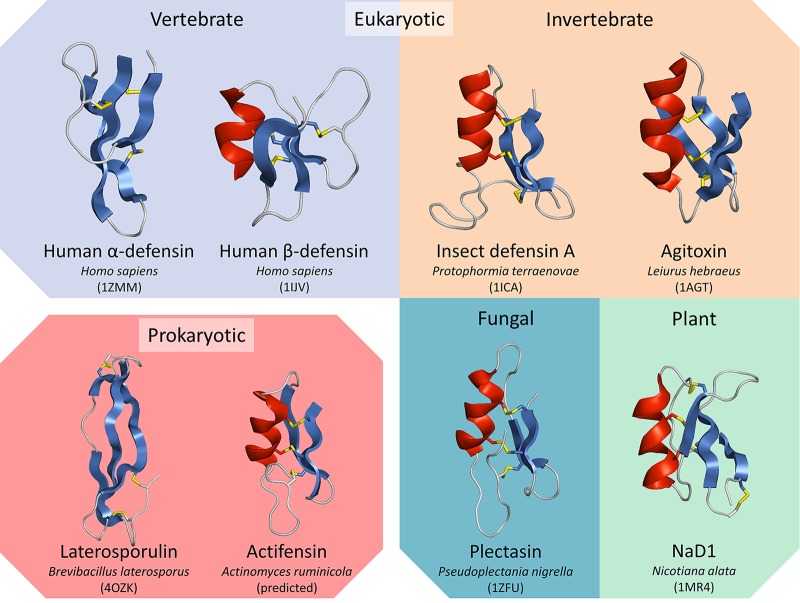
Conserved structures of the defensin peptide superfamily and defensin-like bacteriocins, laterosporulin and actifensin. β sheets are colored blue, α helices are colored red, and disulfide bonds are shown in yellow.

As stated above, the laterosporulins produced by *Brevibacillus* spp. are two structurally defensin-like bacteriocins with broad-spectrum inhibitory activity (5, 6). Their amino acid sequences are 57.6% similar, which is comparable to that for actifensin and its predicted homologs, but share the conserved cysteine residues which form disulfide bridges. Conserved disulfides are characteristic of defensins and are present in vertebrate, invertebrate, plant, fungal defensins, and defensin-like peptides (4). Actifensin has a predicted mass of 4,097.7 Da, but the actual mass is 4,091 ± 1 Da by MALDI-TOF MS. The same discrepancy in predicted and observed masses was noted with laterosporulin, where six hydrogen atoms are lost in the formation of disulfide bonds. We hypothesize that bonds in actifensin likely form in the 1-4, 2-5, and 3-6 formations, similar to that in ascomycete and arthropod C6 defensins (Fig. 6), as the amino acid motifs (C-X_5–12_-C-X_3_-C-X_9–10_-C-X_4–5_-C-X-C) are conserved (5). The structure of laterosporulin10 has been determined to be architecturally similar to human α-defensin, though its disulfide connectivity is homologous to that of β-defensins (Fig. 8) (6). The overall architecture and disulfide connectivity of actifensin are likely to be homologous to those of C6 defensins, consisting of an N-terminal α-helix followed by a two-stranded antiparallel β-sheet stabilized by disulfide bridges (Fig. 8). Interestingly, an actifensin homolog we identify as AfnA from *Actinomyces* sp. oral taxon 171 strain F0337 has had its three-dimensional (3D) structure determined and is publicly available under PDB accession number 2RU0. The peptide labeled actinomycesin is strikingly similar to C6 fungal and arthropod defensins, which have also been characterized (Fig. 6); however, no published material is available regarding its activity, antimicrobial or otherwise. Indeed, two antiparallel beta sheets stabilized by disulfide bonds with an interposed short turn region, previously described as the γ-core motif, are a ubiquitous feature of antimicrobial peptides (35). Actifensin exhibits the highly conserved GXC (positions 26 to 28 in the mature peptide) as do all of its homologs.

CSαβ peptides comprise one of the most widespread families of defensins and defensin-like peptides. A recent publication identified a number of CSαβ sequences in bacterial genomes with potential for antimicrobial, toxin, or signaling activity (27). Of 58 peptides identified within the phylum *Actinobacteria* by Dash et al. (27), 34 were of the genus *Actinomyces*, 24 of which we identified using BLAST with the actifensin propeptide sequence (see Table S2 in the supplemental material). A further 113 bacterial peptide sequences identified by Dash et al. (27) remain to be characterized from a functional perspective and may be a potent source for antimicrobials. Interestingly, a bacterial defensin-like peptide, AdDLP, identified *in silico* was synthesized and recombinantly expressed, and the peptide was found to have anti-*Plasmodium* activity (28). The bacterial CSαβ peptides may be an untapped source of potential applications and have been proposed as the ancestral evolutionary origin of eukaryotic defensins (29).

In the search for novel antimicrobials for application in health and food, genomic and pangenomic approaches are becoming increasingly common (25, 26). These approaches are advantageous in that large amounts of genetic data can be analyzed to identify novel antimicrobials/bacteriocins and can even allow one to “reincarnate” otherwise “dormant” genes (47). However, such analyses are dependent on the ability of programs to predict based on databases of previously identified sequences, and so peptides with novel structures and operons may not be detected. Though a number of bacteriocin operons were found in the *Actinomyces* spp. genomes using BAGEL, actifensin was not identified by genome sequence alone, which highlights the importance of functional screening for antimicrobial compounds in addition to *in silico* screening. By using BLAST, 37 structural genes with homology to actifensin were found in *Actinomyces* spp. along with a single structural gene from a *Corynebacterium* sp. As some CSαβ peptides function as toxins, future applications will require any potential cytotoxic effects to be assayed. We propose that actifensins and the laterosporulins may constitute a new subgroup of class II bacteriocins: the defensin-like bacteriocins. These bacteriocins share only moderate identity to each other but contain highly conserved cysteine residues and are structurally related to eukaryotic defensins.

### Conclusions.

A series of novel defensin-like bacteriocins within the genus *Actinomyces* were identified using an *in silico* pangenomic approach coupled with a functional screen. The bacteriocins represent a potential new class of antimicrobial peptides, defensin-like bacteriocins, which may have widespread applications as antimicrobials in food and human health.

## MATERIALS AND METHODS

### Isolation of bacteria and identification of bacteriocin production.

Samples of raw milk, unpasteurized cheeses, sheep feces, and honey were serially diluted in maximum recovery diluent (Oxoid) and plated on several medium types for the isolation of bacteriocin-producing bacteria: Streptococcus thermophilus selective agar (tryptone, 10.0 g · liter^−1^; sucrose, 10.0 g · liter^−1^; yeast extract, 5.0 g · liter^−1^; K_2_HPO_4_, 2.0 g · liter^−1^; bromocresol purple, 0.03 g · liter^−1^; agar, 15.0 g · liter^−1^) incubated aerobically at 42°C; M17 (Merck) supplemented with 10% (wt/vol) lactose incubated at 30°C aerobically; de Man, Rogosa, and Sharpe (MRS; Difco) agar supplemented with 30 μg · ml^−1^ l-vancomycin hydrochloride incubated at 37°C; MRS adjusted to pH 5.4 incubated at 42°C anaerobically; *Lactobacillus* selective agar (LBS) incubated at 30°C anaerobically; and TOS (transgalactosylated oligosaccharide) agar supplemented with 50 μg · ml^−1^ lithium mupirocin incubated at 37°C anaerobically.

Isolates were subject to an initial bacteriocin production screen by overlaying with 10 ml “sloppy” MRS agar (7.5 g · liter^−1^ agar) tempered to 50°C and seeded with an overnight culture of Lactobacillus delbrueckii subsp. *bulgaricus* LMG 6901 (0.25% [vol/vol]). Cultures which were found to produce distinct zones of inhibition in the agar overlay were cultured in broth for well diffusion assays. For well diffusion assays, 20 ml of sloppy MRS agar seeded with *L. bulgaricus* LMG 6901 as described above was poured and allowed to set, in which 6-mm-wide wells were then bored. Fifty microliters of cell-free supernatant was added to each well, and plates were incubated at 37°C overnight. Zones of inhibition were indicative of antimicrobial activity.

### Bacterial strains, media, reagents.

Strains used in this study and their incubation conditions are listed in Table S3 in the supplemental material. *A. ruminicola* DPC 7226 was routinely maintained in brain heart infusion (BHI) broth (Oxoid) anaerobically at 37°C. Medium reagents were sourced from Sigma-Aldrich (Wicklow, Ireland) unless stated otherwise.

### Purification of actifensin.

*A. ruminicola* DPC 7226 was grown anaerobically and statically at 37°C in 500-ml volumes of BHI broth for 48 h. Following centrifugation, cell-free supernatant was applied to an Econo column containing 30 g Amberlite XAD beads prewashed with Milli-Q water. The column was washed with 300 ml 30% ethanol and 300 ml 2-propanol–0.1% trifluoroacetic acid (TFA) (IPA). IPA was removed by rotary evaporation, and the sample was applied to a 60-ml 10-g Strata-E C_18_ SPE column (Phenomenex, Cheshire, UK) preequilibrated with methanol and water. The column was washed with 60 ml 25% ethanol and then 60 ml IPA.

Centrifuged cells were combined with 100 ml IPA and stirred at room temperature for 3 to 4 h. The resulting suspension was centrifuged, and the cell extract and purified CFS were assayed by MALDI-TOF mass spectrometry to determine the molecular mass of antimicrobial compounds (Axima TOF^2^ MALDI-TOF mass spectrometer; Shimadzu Biotech, Manchester, UK). A MALDI target plate was precoated with α-cyano-4-hydroxycinnamic acid (CHCA) matrix solution, 0.5 μl of the supernatant from the cell extract was then placed on the target, and a final layer of matrix solution was added. Positive-ion linear or reflectron mode was used to detect peptide masses.

### Actifensin characterization.

Characterization was performed using purified bacteriocin. To test protease susceptibility, 100-μl aliquots of 50 μg · ml^−1^ were subjected to treatment with 20 mg · ml^−1^ proteinase K (Sigma-Aldrich) and α-chymotrypsin (Sigma-Aldrich) at 37°C for 3 h, followed by a 10-min incubation at 100°C to denature the enzymes. Fifty-microliter aliquots were assayed on *L. delbrueckii* subsp. *bulgaricus* LMG 6901 indicator plates. Heat stability was determined by 30-min incubations at 60°C, 70°C, 80°C, 90°C, and 100°C and by autoclaving at 121°C for 15 min.

For spectrum of activity, a well diffusion assay was carried out as described above with the strains in in the appropriate medium. Fifty microliters of purified bacteriocin at a concentration of 50 μg · ml^−1^ was added to a well. Following overnight incubation under the appropriate conditions, zones of activity were measured and categorized as no inhibition, weak inhibition (0.5 mm to 2 mm), strong inhibition (2.5 mm to 5 mm), and very strong inhibition (>5 mm). MIC against selected pathogens was assayed as described above, starting at 100 μg · ml^−1^ peptide solution and serially diluted 1:2 to 0.78 μg · ml^−1^.

### Draft genome sequencing.

DNA was extracted using a GenElute bacterial genomic DNA kit (Sigma) and prepared for sequencing using a Nextera XT kit (Illumina) for library preparation. DNA was quantified using a Qubit 2.0 fluorometer. Sequencing was carried out using an Illumina MiSeq platform with paired-end 2 × 300-bp reads by the Teagasc Sequencing Centre, Teagasc Food Research Centre, Moorepark, Fermoy, Ireland. Assembly was performed using tools available on the public server at https://usegalaxy.org (30). Assembly was performed *de novo* using SPADES (version 3.0.0) and resulted in 116 contigs. Contigs were aligned to a reference genome using Mauve (version 20150226, build 10), followed by annotation with RAST (version 2.0). The annotated genome was analyzed for predicted bacteriocin and secondary metabolite production clusters using BAGEL4 (37), and any further annotation was carried out using Artemis genome browser (version 16.0.0).

### BAGEL screen and phylogenetic analysis of *Actinomyces* species.

GenBank and FASTA assemblies of the genus *Actinomyces* were acquired from the NCBI assembly database and screened using BAGEL4 (37). Where available, corresponding 16S rRNA sequences were acquired from the RDP database (38), and where unavailable, *Actinomyces* sp. genomes were subject to analysis using RNAmmer (32). 16S rRNA sequences were aligned using MUSCLE (33), and a phylogram was generated using iTOL (40). The phylogram was then overlaid with the BAGEL screen data.

### Reverse bacteriocin identification, peptide and structure prediction, and homology.

Two hundred micrograms freeze-dried purified peptide was sent for N-terminal amino acid sequencing (AltaBioscience, UK). The resulting 15-residue sequence, GFGXNLITSNPYQXS, was used to search for a bacteriocin structural gene with Artemis genome browser. Following identification of the structural gene, other genomes were searched for genes homologous to the active and propeptide using BLASTp; genes on contigs consisting of less than 5 kbp were excluded. Additional actifensin homologs were identified from the study by Dash et al. (27) among 147 nonredundant bacterial CSαβ peptide sequences (27). Alignments were generated using Clustal Omega (41) and visualized with Jalview (42). Structural modeling was performed using SWISSMODEL (43) online software, and structural images were generated using PyMOL (44).

### Data availability.

Genomic data analyzed in this study were deposited in GenBank/EMBL under accession number SPKK00000000 and are publicly available from the NCBI database at https://www.ncbi.nlm.nih.gov/.

## Supplementary Material

Supplemental file 1

Supplemental file 2

Supplemental file 3

Supplemental file 4
