# Supplementary material for: Actinomyces Produces Defensin-Like Bacteriocins (Actifensins) with a Highly Degenerate Structure and Broad Antimicrobial Activity
Source: J Bacteriol. 2020 Jan 29;202(4):e00529-19. doi: 10.1128/JB.00529-19 (PMC6989792; doi:10.1128/JB.00529-19)
Supplement: Supplemental file 4 [file JB.00529-19-s0004.pdf]

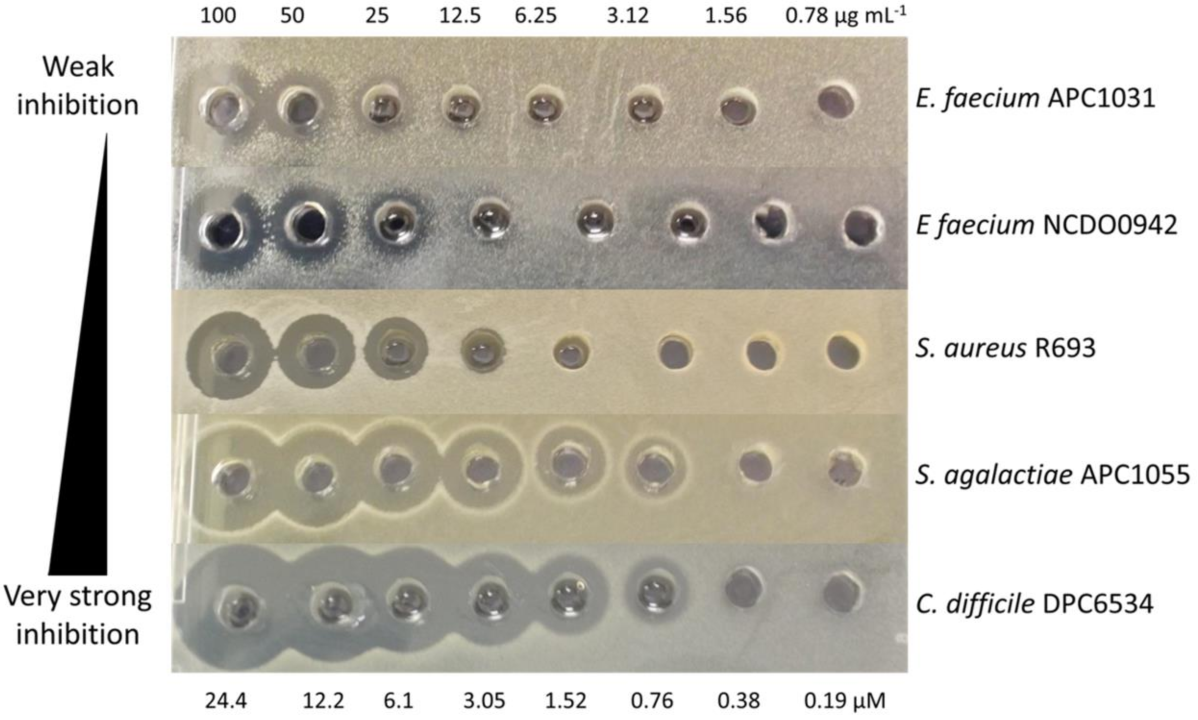

Supplementary Figure 1. Minimum inhibitory concentration of actifensin peptide against Gram positive pathogens determined by well diffusion assay.

Phylum	Species	Per. Identity	Description	
Actinobacteria	*A. ruminicola*	-	AfnA	--GFGCNLITSNPYQCSNHCKSV-GYRGGYCKLRTVCTCY----------
Ascomycota	*Helicocarpus griseus*	52.26	hypothetical protein	MRFSTVFAVVSALSMTALALPSPVTEDVNLAE-------REAAPEPMPEELVAAFTKLGERSLEGEEDN----VIAKRGFGCTIWGGNDKPCHRHCKSIKGYKGGYCKVGGVCKCY----------
Ascomycota	*Blastomyces percursus*	57.89	hypothetical protein	MRLSAVFAIISALSMTALAIPAPAPEDLDIAEATADLATRDAPVEAIPDDFVGDLAGLNDDDDDDDEDERPAHALQKRGWGCNIFGGNDYRCHRHCKSIRGYKGGYCKLGGICKCY----------
Ascomycota	*Blastomyces silverae*	57.89	hypothetical protein	MRFSAVFAIISALSMTALAIPAPAPEDLDIAEATADLAARDARMGAIPDDFAGDLAGLDDDDDDDDEDENPARTLQKRGWGCNIFGGNDYRCHRHCKSIKGYKGGYCKLGGICKCY----------
Ascomycota	*Blastomyces gilchristii*	57.89	conserved hypothetical protein	MRFSAVFAIISALSMTALAIPAPAPEDLDIAEATADLAARDAPVEAIPDDFAGDVSGLDDEDD-----ENSAGALQKRGWGCNIFGGNDYRCHRHCKSISGYKGGYCKLGGICKCY----------
Ascomycota	*Blastomyces dermatitidis*	57.89	hypothetical protein	MRFSAVFAIISALSMTALAIPVPAPEDLDIAEATADLAARDAPVEAIPDDFAGDVSGLDDDDDDDDEDENSAGALQKRGWGCNIFGGNDYRCHRHCKSISGYKGGYCKLGGICKCY----------
Ascomycota	*Blastomyces dermatitidis*	57.89	hypothetical protein	MRFSAVFAIISALSMTALAIPAPAPEDLDIAEATADLAARDAPVEAIPDDFAGDVSGLDDDDDDDDEDENSAGALQKRGWGCNIFGGNDYRCHRHCKSISGYKGGYCKLGGICKCY----------
Ascomycota	*Emmonsia crescens*	55.26	hypothetical protein	MRFSAIFAIISALSMTALAMPAPAPEDFGIAEAAADLAARNAPADAIPDDFAGDLAGMDDDDDDDDY-ENSVGSLQKRGWGCTIFGGNDSRCHRHCKSIRGYRGGYCKLGGICKCY----------
Ascomycota	*Emmonsia* sp.	55.26	hypothetical protein	MRASAIFAIISALSMTTLAMPAAAPEDFDIAAATADLAARGAPAEAIPDDFAGDLAGLDDDDDDDDEND-SAGVLQKRGWGCTIFGGNDSRCHRHCKSIRGYRGGYCKLGGICKCY----------
Ascomycota	*Emergomyces pasteurianus*	55.26	hypothetical protein	MRVSAILAIISALSMTALAIPAPAPEDFDIAEATADLAARDAPAEAVPDDFAGDLAGLDDDDDDDDY-ENSAGVLQKRGWGCTIFGGNDSRCHKHCKSIRGYRGGYCKLGGICKCY----------
Mollusca	*Ruditapes philippinarum*	60.53	defensin	MKMMIVFTV---LFLAAMILP-----DVDA--GFGCP----NDYSCSNHCRDSIGCRGGYCKYHVICTCYGCKKRRSIQE

Supplementary Figure 2. Eukaryotic sequences with homology to afnA identified using BLASTp. Residues with 100% identity are highlighted.

Genus_species_strain_peptide		1	2	3	4	5	6	7	8	9	10	11	12	13	14	15	16	17	18	19	20	21	22	23	24	25	26	27	28	29	30	31	32	33	34	35	36	37	38	39	40	41	42	43	44	45	46	47	48	49
Corynebacterium_sp_HMSC06D04_afnA	1	100	33	30	32	32	32	32	32	31	42	42	42	41	41	34	40	40	34	34	34	35	34	35	35	39	42	45	45	45	41	41	33	47	44	53	45	45	45	40	40	48	42	48	48	48	48	39	44	42
Actinomyces_oris_MMRCO6_1_C75_afnA2	2	33	100	94	96	96	87	88	88	87	44	44	44	51	51	35	43	43	43	43	43	43	43	42	42	38	46	40	39	39	36	38	46	43	45	42	42	42	44	39	39	40	37	39	39	41	41	34	38	44
Actinomyces_oris_CCUG_34286_afnA7	3	30	94	100	99	99	87	85	85	86	47	47	47	53	53	36	43	43	45	45	45	45	45	43	43	38	47	42	42	42	38	42	48	46	46	44	45	45	47	41	41	43	40	42	42	44	44	34	41	47
Actinomyces_oris_S64C_afnA5	4	32	96	99	100	100	88	84	84	84	47	47	47	53	53	36	43	43	45	45	45	45	45	43	43	38	47	42	42	42	38	42	48	46	46	44	45	45	47	41	41	43	40	42	42	44	44	34	41	47
Actinomyces_oris_S24V_afnA5	5	32	96	99	100	100	88	84	84	84	47	47	47	53	53	36	43	43	45	45	45	45	45	43	43	38	47	42	42	42	38	42	48	46	46	44	45	45	47	41	41	43	40	42	42	44	44	34	41	47
Actinomyces_sp_oral_taxon_171_F0337_afnA4	6	32	87	87	88	88	100	88	88	86	48	48	48	53	53	35	45	45	43	43	43	43	43	42	42	38	46	42	39	39	38	40	45	45	43	42	45	45	45	41	41	42	38	40	40	43	43	35	41	47
Actinomyces_oris_MMRCO6_1_C50_afnA4	7	32	88	85	84	84	88	100	100	100	47	47	47	53	53	35	43	43	43	43	43	43	43	42	42	38	46	42	36	36	38	38	45	45	43	42	44	44	44	39	39	40	38	40	40	43	43	34	40	46
Actinomyces_oris_MMRCO6_1_C50_afnA3	8	32	88	85	84	84	88	100	100	100	47	47	47	53	53	35	43	43	43	43	43	43	43	42	42	38	46	42	36	36	38	38	45	45	43	42	44	44	44	39	39	40	38	40	40	43	43	34	40	46
Actinomyces_sp_HMSC075C01_afnA3	9	31	87	86	84	84	86	100	100	100	46	46	46	52	52	34	40	40	42	42	42	42	42	42	42	37	44	42	35	35	38	38	45	44	44	43	44	44	44	39	39	40	38	40	40	43	43	33	40	44
Actinomyces_oris_MMRCO6_1_C50_afnA1	10	42	44	47	47	47	48	47	47	46	100	100	98	64	64	45	49	49	49	49	49	51	49	52	52	53	58	51	48	48	50	52	58	57	57	58	56	56	52	56	56	55	56	63	63	61	61	42	48	59
Actinomyces_sp_HMSC075C01_afnA1	11	42	44	47	47	47	48	47	47	46	100	100	98	64	64	45	49	49	49	49	49	51	49	52	52	53	58	51	48	48	50	52	58	57	57	58	56	56	52	56	56	55	56	63	63	61	61	42	48	59
Actinomyces_sp_oral_taxon_171_F0337_afnA1	12	42	44	47	47	47	48	47	47	46	98	98	100	64	64	45	49	49	49	49	49	51	49	52	52	53	58	51	48	48	50	52	58	57	57	58	56	56	52	56	56	55	56	63	63	61	61	42	48	58
Actinomyces_sp_Chiba101_afnA1	13	41	51	53	53	53	53	53	53	52	64	64	64	100	100	41	45	45	42	42	42	40	40	40	40	42	54	40	40	40	42	48	46	54	51	56	52	52	53	45	45	51	47	54	54	51	51	38	45	54
Actinomyces_denticolens_PA_afnA1	14	41	51	53	53	53	53	53	53	52	64	64	64	100	100	41	45	45	42	42	42	40	40	40	40	42	54	40	40	40	42	48	46	54	51	56	52	52	53	45	45	51	47	54	54	51	51	38	45	54
Actinomyces_sp_2119_afnA1	15	34	35	36	36	36	35	35	35	34	45	45	45	41	41	100	46	46	52	52	52	52	52	54	54	55	61	58	45	45	48	47	49	54	49	49	47	47	49	50	50	52	47	49	49	50	50	41	47	53
Actinomyces_sp_Chiba101_afnA2	16	40	43	43	43	43	45	43	43	40	49	49	49	45	45	46	100	100	61	61	61	63	63	60	60	52	61	61	49	49	48	53	53	59	55	55	55	55	52	46	46	53	52	48	48	52	52	46	43	49
Actinomyces_denticolens_PA_afnA2	17	40	43	43	43	43	45	43	43	40	49	49	49	45	45	46	100	100	61	61	61	63	63	60	60	52	61	61	49	49	48	53	53	59	55	55	55	55	52	46	46	53	52	48	48	52	52	46	43	49
Actinomyces_oris_MMRCO6_1_C50_afnA2	18	34	43	45	45	45	43	43	43	42	49	49	49	42	42	52	61	61	100	100	99	94	93	91	91	57	58	55	49	49	57	56	53	53	53	55	52	52	51	48	48	55	51	45	45	51	51	45	49	58
Actinomyces_sp_HMSC075C01_afnA2	19	34	43	45	45	45	43	43	43	42	49	49	49	42	42	52	61	61	100	100	99	94	93	91	91	57	58	55	49	49	57	56	53	53	53	55	52	52	51	48	48	55	51	45	45	51	51	45	49	58
Actinomyces_sp_oral_taxon_171_F0337_afnA2	20	34	43	45	45	45	43	43	43	42	49	49	49	42	42	52	61	61	99	99	100	96	94	93	93	57	58	55	49	49	57	56	53	53	53	55	52	52	51	48	48	55	51	45	45	51	51	45	49	58
Actinomyces_oris_CCUG_34286_afnA5	21	35	43	45	45	45	43	43	43	42	51	51	51	40	40	52	63	63	94	94	96	100	96	96	96	57	61	58	51	51	58	58	55	55	55	57	54	54	51	48	48	56	52	47	47	52	52	46	49	61
Actinomyces_oris_CCUG_34286_afnA2	22	34	43	45	45	45	43	43	43	42	49	49	49	40	40	52	63	63	93	93	94	96	100	97	97	55	58	55	49	49	60	56	53	53	53	55	52	52	49	46	46	53	51	45	45	51	51	46	48	58
Actinomyces_oris_S64C_afnA2	23	35	42	43	43	43	42	42	42	42	52	52	52	40	40	54	60	60	91	91	93	96	97	100	100	57	60	57	49	49	62	58	56	53	56	59	54	54	49	49	49	55	52	47	47	52	52	46	49	60
Actinomyces_oris_S24-V_afnA2	24	35	42	43	43	43	42	42	42	42	52	52	52	40	40	54	60	60	91	91	93	96	97	100	100	57	60	57	49	49	62	58	56	53	56	59	54	54	49	49	49	55	52	47	47	52	52	46	49	60
Actinomyces_sp_2119_afnA2	25	39	38	38	38	38	38	38	38	37	53	53	53	42	42	55	52	52	57	57	57	57	55	57	57	100	70	63	48	48	43	48	52	52	55	53	52	52	47	51	51	58	49	49	49	51	51	45	45	54
Actinomyces_sp_2119_afnA3	26	42	46	47	47	47	46	46	46	44	58	58	58	54	54	61	61	61	58	58	58	61	58	60	60	70	100	69	52	52	49	52	55	57	54	53	52	52	59	46	46	60	51	51	51	56	56	46	49	61
Actinomyces_sp_2129_afnA	27	45	40	42	42	42	42	42	42	42	51	51	51	40	40	58	61	61	55	55	55	58	55	57	57	63	69	100	66	66	52	53	53	58	61	61	55	55	54	51	51	58	57	50	50	52	52	46	52	55
Actinomyces_sp_oral_taxon_849_str_F0330_afn	28	45	39	42	42	42	39	36	36	35	48	48	48	40	40	45	49	49	49	49	49	51	49	49	49	48	52	66	100	100	48	55	47	50	56	57	52	52	49	48	48	55	51	47	47	48	48	48	51	52
Actinomyces_johnsonii_F0542_afnA	29	45	39	42	42	42	39	36	36	35	48	48	48	40	40	45	49	49	49	49	49	51	49	49	49	48	52	66	100	100	48	55	47	50	56	57	52	52	49	48	48	55	51	47	47	48	48	48	51	52
Actinomyces_naeslundii_S44D_afnA	30	41	36	38	38	38	38	38	38	38	50	50	50	42	42	48	48	48	57	57	57	58	60	62	62	43	49	52	48	48	100	64	44	44	48	55	48	48	51	42	42	55	48	46	46	48	48	45	49	61
Actinomyces_sp_oral_taxon_414_F0588_afnA	31	41	38	42	42	42	40	38	38	38	52	52	52	48	48	47	53	53	56	56	56	58	56	58	58	48	52	53	55	55	64	100	52	52	55	59	58	58	61	45	45	61	54	52	52	55	55	48	54	60
Actinomyces_sp_oral_taxon_171_F0337_afnA3	32	33	46	48	48	48	45	45	45	45	58	58	58	46	46	49	53	53	53	53	53	55	53	56	56	52	55	53	47	47	44	52	100	68	74	61	63	63	52	60	60	54	62	60	60	65	65	37	45	48
Actinomyces_oris_CCUG_34286_afnA3	33	47	43	46	46	46	45	45	45	44	57	57	57	54	54	54	59	59	53	53	53	55	53	53	53	52	57	58	50	50	44	52	68	100	78	80	81	81	56	67	67	64	72	76	76	78	78	46	51	52
Actinomyces_oris_MMRCO6_1_C75_afnA1	34	44	45	46	46	46	43	43	43	44	57	57	57	51	51	49	55	55	53	53	53	55	53	56	56	55	54	61	56	56	48	55	74	78	100	100	85	85	53	67	67	60	72	67	67	72	72	49	55	51
Actinomyces_sp_HMSC075C01_afnA4_	35	53	42	44	44	44	42	42	42	43	58	58	58	56	56	49	55	55	55	55	55	57	55	59	59	53	53	61	57	57	55	59	61	80	100	100	93	93	57	60	60	67	67	67	67	67	67	56	60	56
Actinomyces_oris_S64C_afnA3	36	45	42	45	45	45	45	44	44	44	56	56	56	52	52	47	55	55	52	52	52	54	52	54	54	52	52	55	52	52	48	58	63	81	85	93	100	100	57	64	64	62	72	70	70	72	72	48	53	52
Actinomyces_oris_S24V_afnA3	37	45	42	45	45	45	45	44	44	44	56	56	56	52	52	47	55	55	52	52	52	54	52	54	54	52	52	55	52	52	48	58	63	81	85	93	100	100	57	64	64	62	72	70	70	72	72	48	53	52
Actinomyces_ruminicola_DPC7226_afnA	38	45	44	47	47	47	45	44	44	44	52	52	52	53	53	49	52	52	51	51	51	51	49	49	49	47	59	54	49	49	51	61	52	56	53	57	57	57	100	46	46	77	56	58	58	62	62	48	64	65
Actinomyces_oris_S64C_afnA1	39	40	39	41	41	41	41	39	39	39	56	56	56	45	45	50	46	46	48	48	48	48	46	49	49	51	46	51	48	48	42	45	60	67	67	60	64	64	46	100	100	55	66	72	72	72	70	52	55	49
Actinomyces_oris_S24V_afnA1	40	40	39	41	41	41	41	39	39	39	56	56	56	45	45	50	46	46	48	48	48	48	46	49	49	51	46	51	48	48	42	45	60	67	67	60	64	64	46	100	100	55	66	72	72	72	70	52	55	49
Actinomyces_sp_CtC_72_afnA	41	48	40	43	43	43	42	40	40	40	55	55	55	51	51	52	53	53	55	55	55	56	53	55	55	58	60	58	55	55	55	61	54	64	60	67	62	62	77	55	55	100	62	71	71	74	72	54	63	69
Actinomyces_oris_CCUG_34286_afnA6	42	42	37	40	40	40	38	38	38	38	56	56	56	47	47	47	52	52	51	51	51	52	51	52	52	49	51	57	51	51	48	54	62	72	72	67	72	72	56	66	66	62	100	74	74	80	80	46	50	49
Actinomyces_oris_S64C_afnA4	43	48	39	42	42	42	40	40	40	40	63	63	63	54	54	49	48	48	45	45	45	47	45	47	47	49	51	50	47	47	46	52	60	76	67	67	70	70	58	72	72	71	74	100	100	91	90	45	46	51
Actinomyces_oris_S24V_afnA4	44	48	39	42	42	42	40	40	40	40	63	63	63	54	54	49	48	48	45	45	45	47	45	47	47	49	51	50	47	47	46	52	60	76	67	67	70	70	58	72	72	71	74	100	100	91	90	45	46	51
Actinomyces_oris_CCUG_34286_afnA4	45	48	41	44	44	44	43	43	43	43	61	61	61	51	51	50	52	52	51	51	51	52	51	52	52	51	56	52	48	48	48	55	65	78	72	67	72	72	62	72	72	74	80	91	91	100	99	47	50	53
Actinomyces_oris_CCUG_34286_afnA1	46	48	41	44	44	44	43	43	43	43	61	61	61	51	51	50	52	52	51	51	51	52	51	52	52	51	56	52	48	48	48	55	65	78	72	67	72	72	62	70	70	72	80	90	90	99	100	47	50	53
Actinomyces_naeslundii_Pn6N_afnA	47	39	34	34	34	34	35	34	34	33	42	42	42	38	38	41	46	46	45	45	45	46	46	46	46	45	46	46	48	48	45	48	37	46	49	56	48	48	48	52	52	54	46	45	45	47	47	100	55	51
Actinomyces_succiniciruminis_AM4_afnA2	48	44	38	41	41	41	41	40	40	40	48	48	48	45	45	47	43	43	49	49	49	49	48	49	49	45	49	52	51	51	49	54	45	51	55	60	53	53	64	55	55	63	50	46	46	50	50	55	100	65
Actinomyces_succiniciruminis_AM4_afnA1	49	42	44	47	47	47	47	46	46	44	59	59	58	54	54	53	49	49	58	58	58	61	58	60	60	54	61	55	52	52	61	60	48	52	51	56	52	52	65	49	49	69	49	51	51	53	53	51	65	100
				30	40	50	60	70	80	90	100																																							

Supplementary Figure 3. Percent-identity matrix of actifensin (Actifensin_ruminicola_DPC7226_afnA) mature peptide amino acid sequence and homologous afnA sequences.

Supplementary Table 1. *Actinomyces* genomes used in in silico screen.

Supplmentary Table 2. afnA mature sequences and source genomes.

Supplementary Table 3. Growth conditions of indicator species used in this study.

References

1. Dash TS, Shafee T, Harvey PJ, Zhang C, Peigneur S, Deuis JR, Vetter I, Tytgat J, Anderson MA, Craik DJ. 2019. A centipede toxin family defines an ancient class of CSαβ defensins. Structure 27:315-326. e7.
